# Deciphering the Stromal and Hematopoietic Cell Network of the Adventitia from Non-Aneurysmal and Aneurysmal Human Aorta

**DOI:** 10.1371/journal.pone.0089983

**Published:** 2014-02-27

**Authors:** Charles-Antoine Dutertre, Marc Clement, Marion Morvan, Knut Schäkel, Yves Castier, Jean-Marc Alsac, Jean-Baptiste Michel, Antonino Nicoletti

**Affiliations:** 1 INSERM (Institut National de la Santé et de la Recherche Médicale) U698, Hemostasis, Bio-engineering and Cardiovascular Remodeling, Hôpital Bichat, Paris, France; 2 Université Denis Diderot, Paris, France; 3 Department of Dermatology, University Hospital Heidelberg, Germany; 4 AP-HP, Service de chirurgie Vasculaire et Thoracique, Hôpital Bichat, Paris, France; 5 AP-HP, Service de chirurgie cardiaque et vasculaire, Hopital Européen Georges Pompidou, Faculté de Médecine René Descartes, Université Paris 5, Paris, France; 6 DHU Fire, Paris, France; University Hospital Medical Centre, Germany

## Abstract

Aneurysm is associated to a complex remodeling of arteries that affects all their layers. Although events taking place in the intima and the media have received a particular attention, molecular and cellular events taking place in the adventitia have started to be deciphered only recently. In this study, we have precisely described the composition and distribution of stromal and hematopoietic cells in human arterial adventitia, both at steady state and in the setting of aortic aneurysm. Using polychromatic immunofluorescent and flow cytometry analyses, we observed that unlike the medial layer (which comprises mostly macrophages and T cells among leukocytes), the adventitia comprises a much greater variety of leukocytes. We observed an altered balance in macrophages subsets in favor of M2-like macrophages, an increased proliferation of macrophages, a greater number of all stromal cells in aneurysmal aortas. We also confirmed that in this pathological setting, adventitia comprised blood vessels and arterial tertiary lymphoid organs (ATLOs), which contained also M-DC8^+^ dendritic cells (slanDCs) that could participate in the induction of T-cell responses. Finally, we showed that lymphatic vessels can be detected in aneurysmal adventitia, the functionality of which will have to be evaluated in future studies. All together, these observations provide an integrative outlook of the stromal and hematopoietic cell network of the human adventitia both at steady state and in the context of aneurysm.

## Introduction

Inflammatory mechanisms taking place in the intimal region of atherothrombotic arteries have been extensively studied. They are intimately linked to the clinical manifestation of atherosclerosis. Alterations in the medial layer are also well documented [1]. However, major biological processes that take place in the adventitia close to thrombotic regions could participate in the physiopathology of atherothrombotic disorders. Indeed, adventitial inflammation and the presence of lymphoid-like cell clusters called arterial tertiary lymphoid organs (ATLOs), first observed years ago [2]–[4], have been revisited recently [1], [5]–[9]. These structures could support the local maturation of athero-modulating immune effectors.

We found that these lymphoid structures were prominently developed around aneurysmal aortas and, in the present study, we have established a precise cartography of stromal and hematopoietic cells comprised in fresh periarterial human tissues with active atherothrombotic complications. In particular, we wished to determine the presence or not of cells known to be involved both in the formation, the maintenance and the function of the ATLOs.

It is now well established that particular stromal cells called lymphoid tissue organizer cells (LTo) collaborate with lymphoid tissue inducer cells (LTi) of hematopoietic origin for the development of secondary lymphoid organs (SLOs) during embryogenesis, and for the formation of TLOs in the context of chronic inflammatory diseases [10]–[12]. These stromal LTo were described as expressing various molecules such as gp38 (podoplanin), MadCAM1, ICAM1 or VCAM1 [13], [14], and following their activation, promote the recruitment of lymphocytes thus organizing the future lymphoid structure. The nature of LTi cells in the context of TLO genesis remains more of a debate and could differ depending on the tissue and the pathological setting [10]. A major feature of LTi cells is their production of TNFα or Lymphotoxin α (LTα), which allow them to activate and cross-talk with LTo cells leading to TLO genesis. LTi cells, that could account for the formation and/or maintenance of ATLOs, could be M1-polarized TNFα-producing macrophages [15]–[17], or a discrete cellular subset called slanDC (CD14^lo^slan/M-DC8^+^) [18]. SlanDC were recently shown to be responsible for TNFα overproduction during chronic HIV infection [19]. They were detected in T-cell areas of mucosa-associated lymphoid tissue [20] and in lesional tissues from various chronic inflammatory diseases [20]–[22].

Other cell subsets have an important role in SLOs and TLOs. Among (non-hematopoietic) stromal cells, fibroblastic reticular cells (FRC, gp38^+^CD44^+^CD31^−^) are essential for their maintenance, and follicular dendritic cells (FDC, gp38^int^CD21^hi^), which present native antigens to naive B cells, participate in the mounting of adaptive B-cell responses. Among hematopoietic cells, besides B and T cells, dendritic cells, which present antigens and provide proper costimulatory signals, are mandatory for mounting efficient adaptive immune responses.

First, we have evaluated, by polychromatic immunofluorescent analyses of arterial cross-sections, the localization of major leukocyte and stromal cell subsets. We could detect, specifically in the adventitia of aneurysmal aortas, nodular lymphoid aggregates displaying all the structural features [FRC network, blood vessels layered by High Endothelial Vein (HEV)-like cells, lymphatics] and cell composition (T/B cell compartments, FDC) of ATLOs. In order to provide a quantitative analysis of the cell composition of the vessel wall, we used polychromatic flow cytometry that allows the precise identification of multiple cellular subsets from a limited number of cells, as it is often the case when working with human rare and precious tissue samples. By adapting tissue dissection and a specific digestion protocol adapted from Fletcher et al. [14] we could release cells comprised in the different layers of arterial tissue samples. We built two multi-color panels to identify simultaneously multiple leukocyte or stromal cell subsets in deep layers of human non-aneurysmal and aneurysmal aortas from various arterial territories, which, to our knowledge, has never been done. Most non-aneurysmal aortas presenting initial atheromatous lesions were used as controls.

Our results show that the adventitia is a remarkably complex layer far from being only composed of fibroblasts. Instead, it is composed of an important diversity of cells of hematopoietic and non-hematopoietic (stromal) origin, in both aneurysmal and non-aneurysmal aortas. While their proportions are different in the two types of aortas, the fact that they are found in the absence of ATLOs and in non-aneurysmal aortas indicates that the adventitial layer is pre-disposed and prone for the lymphoid neogenesis that takes place under vascular chronic inflammatory stimulation.

## Materials and Methods

### Tissue Samples and Cell Isolation

Aneurysmal aortas (n = 6) were obtained from patients undergoing surgery and enrolled in the RESAA protocol (REflet Sanguin de l’évolutivité des Anévrismes de l’Aorte abdominale, Comité Consultatif de Protection des Personnes dans la Recherche Biomedicale, CCPRB Paris-Cochin, approval no 2095). Written informed consent were obtained from patients. Non-aneurysmal aortas (n = 4) were sampled from deceased organ donors with the authorization of the French Biomedicine Agency (PFS 09-007). The data were analyzed anonymously. Inflammatory aneurysms, pseudoaneurysms or dissected aneurysms were not included in this study. Clinical and observational data for all tissues analyzed are reported in Table 1. After intraluminal thrombus removal, the media (aneurysmal, n = 2, non-aneurysmal, n = 2) was separated from the adventitia following a dissection protocol routinely used in the laboratory. Our previous studies have repeatedly shown that we can obtain the medial and adventitial layers with minimal cross-contamination [23], [24]. Each layer was then cut into small pieces (1–2 mm^3^) that were placed in enzyme mix [DMEM (Gibco) containing Dispase I (2.4 mg/mL, Roche), Collagenase P (0.6 mg/mL, Roche) and DNase I (Invitrogen, 0.3 mg/mL)] for adventitia and with also Elastase 0.1% (Worthington) for media pieces. Tubes were incubated at 37°C and inverted every 5 min during the 20 min incubation (first digestion round). The enzyme mix, containing cells, was then separated from remaining pieces and added to ice-cold PBS 1x on ice. Remaining tissue pieces were placed in fresh enzyme mix for a second digestion round (37°C, 20 min) and enzyme mix was pooled with that of the first digestion round. A third digestion round was carried out and the pooled recovered enzyme mix was centrifuged to recover cells that were washed two times in ice-cold PBS 1x.

**Table 1:** Tissue samples and clinical data from patients and donors.

Tissue type	Aneurysm	Leukocyte panel	Stromal cell panel	Age	Sex	aortic diameter (mm)
Abdominal aorta	NO	YES	NO	12	M	
Abdominal aorta	NO	YES	YES	47	M	
Abdominal aorta	NO	YES	NO	47	M	
Abdominal aorta	NO	YES	YES	57	M	
Non-aneurysmal (n, median)		4	2	47	0F/4M	
Thoracic aorta	YES	YES	NO	64	M	39
Abdominal aorta	YES	YES	NO	61	M	51
Abdominal aorta	YES	NO	YES	62	M	49
Abdominal aorta	YES	YES	YES	66	F	47
Abdominal aorta	YES	NO	YES	82	M	65
Abdominal aorta	YES	YES	NO	85	M	60
Aneurysmal (n, median)		4	3	65	1F/5M	50

### Polychromatic Flow Cytometric Analyses

Total cellular suspensions were incubated for 30 min at 4°C with Live/Dead Yellow dye (Invitrogen – Life Technologies) in PBS. 5% heat-inactivated human AB serum (serum-AB, Abcys) was added for an extra 15 min at 4°C. Next, cell membranes were labeled for 30 min at 4°C with antibodies diluted in PBS with 2% FCS and 2 mM EDTA. For intracellular αSMA and Vybrant Violet staining in stromal cell analyses, cells were fixed and permeabilized with BD Cytofix/Cytoperm kit (BD Biosciences) following manufacturer’s instructions and incubated with the anti-αSMA monoclonal antibody (45 min, +4°C). Vybrant Violet (Invitrogen – Life Technologies) was added for the last 5 minutes following manufacturer’s instructions. Cells were then washed and events acquired using a BD FACS LSRII (BD Biosciences). The configuration of our BD FACS LSRII is shown in Table I in the online-only Data Supplement. All analyses were carried out with the BD FACSDiva (BD Biosciences) software. The absolute number of cells/g tissue was calculated by dividing the total count of each cellular subset by the weight of the tissue sample.

### Immunofluorescent and Histochemical Analyses on Tissue Cross-sections

Paraffin-embedded human arterial cross-sections were deparaffinized in toluene (3×2 min), followed by absolute ethanol (3×2 min) and distilled H_2_O (2 min), and unmasked by heat treatment in antigen retrieval reagent (R&D Systems, 20 min, 95°C). After two washes in PBS 1x, endogenous avidin/biotin were blocked with Biotin-Blocking System (Dako) following manufacturer’s instructions and saturated 30 min with PBS 1x containing 2.5% IgG-free BSA (Jackson Immunoresearch) and 100 mM glycine (VWR). Primary antibodies diluted in PBS 1x, 2.5% IgG-free BSA, 0.05% fish gelatin (Sigma-Adlrich) were then added on cross-section (overnight, 4°C). Following two washes with PBS 1x, secondary antibodies diluted in the same buffer were added (1 hour, RT). Following two washes with PBS 1x, fluorochrome-coupled streptavidin diluted in the same buffer was added (1 hour, RT). After three washes with PBS 1x, Hoechst 33342 (1/1000, Molecular Probes) diluted in the same buffer was added to stain nuclei (5 min, RT). After three washes, immunostained slides were cover-mounted with Prolong Gold antifade reagent (Invitrogen – Life Technologies). The fluorescence was detected with a Zeiss Axio Observer.Z1 microscope equipped with an ORCA-R2 C10600 digital CCD camera (Hamamatsu) and the Zen 2012 image capture software. Images were acquired using the Plan-Apochomat 40x/1.3 Oil DIC M27 (df = 0.2 mm) objective of the microscope, and were analyzed using Photoshop CS4 software (vers.10.0.2, Adobe).

### Antibodies used in Flow Cytometric and Cross-section Immunofluorescent Experiments

The antibodies used for FACS analyses were all mouse anti-human mAbs and were all designed and validated for flow cytometry. For the leukocyte panel, the following antibodies were used: anti-human CD45-V500 (clone HI30, 1/20), anti-human CD15-PE-CF594 (clone HI98, 1/15), anti-human HLA-DR-APC-H7 (clone G46-6, 1/15), anti-human CD8-PerCP (clone SK1, 1/10) all from BD Biosciences; anti-human CD14-BrilliantViolet605 (clone M5E2, 1/15), anti-human CD123-BrilliantViolet650 (clone 6H6, 1/10), anti-human CD3-BrilliantViolet711 (clone OKT3, 1/10), anti-human CD19-BrilliantViolet785 (clone HIB19, 1/80), anti-human CD1c (BDCA-1)-PE-Cy7 (clone L161, 1/20) all from Biolegend; anti-human slan (M-DC8)-FITC (clone DD-1, 1/20), anti-human CD163-Vioblue (clone GHI/61.1, 1/10), anti-human CD141 (BDCA-3)-APC (clone AD5-14H12, 1/100) all from Miltenyi; anti-human CD206-PerCP-eFluor710 (eBioscience, clone 19.2, 1/10) and anti-human MerTK-PE (R&D Systems, clone 125518, 1/10).

For the stromal cells panel, the following antibodies were used: anti-human CD45-eFluor605 (clone HI30, 1/10), anti-human CD31-APC-eFluor780 (clone WM59, 1/15), anti-human podoplanin (gp38)-PerCP-eFluor710 (clone NZ-1.3, 1/15) all from eBioscience; anti-human CD44-BrilliantViolet785 (clone IM7, 1/15), anti-human CD21-PE-Cy7 (clone Bu32, 1/15) both from Biolegend; anti-human MadCAM1-APC (clone 683715, 1/15), anti-human αSMA-AlexaFluor700 (clone 1A4, 1/20) both from R&D Systems; anti-human VCAM1 (CD106)-FITC (clone 51-10C9, 1/10), anti-human ICAM1 (CD54)-PE (clone HA58, 1/15) both from BD Biosciences.

For immunofluorescent staining on paraffin-embedded tissue cross-sections, the following antibodies were used: anti-human CD31 (mouse IgG1, clone JC70A, Dako, 1/20), anti-human CD20 (mouse IgG1, clone L-26, Dako, 1/200), anti-human CD206 (mouse IgG1, clone 5C11, Abcam, 1/100) and isotype control mouse IgG (Dako, 5 µg/mL) all followed by biotin-coupled goat F(ab)’_2_ anti-mouse IgG (BD Biosciences, 1/200) and AlexaFluor546-coupled streptavidin (Invitrogen – Life Technologies, 1/1000); anti-human podoplanin (gp38) (rat IgG2a, clone NZ-1, Angiobio, 1/200) and isotype control rat IgG (BD Biosciences, 2.5 µg/mL) both followed by AlexaFluor488-coupled goat F(ab)’_2_ anti-rat IgG(Fcγ fragment specific) (Jackson ImmunoResearch, 1/1000); anti-human CD3 (rabbit IgG, polyclonal, Dako, 1/50), anti-human CD14 (rabbit IgG, clone EPR3653, Abcam, 1/250) and isotype control rabbit Ig (Dako, 10 µg/mL) all followed by Dylight649-coupled goat F(ab)’_2_ anti-rabbit IgG (Jackson ImmunoResearch, 1/400); anti-human slan/M-DC8 (mouse IgM, clone DD-2, 1/30 provided by Dr. Knut Schäkel), anti-human CD15 (mouse IgM, clone HI98, Biolegend, 1/100) and isotype control mouse IgM (BD Biosciences, 5 µg/mL) all followed by AlexaFluor488-coupled goat F(ab)’_2_ anti-mouse IgM (Jackson ImmunoResearch, 1/400).

### Statistical Analysis

Results are given as medians. Mann-Whitney test was used to compare non-aneurysmal and aneurysmal groups. Differences were defined as statistically significant when p<0.05. This non-parametric test was performed using GraphPad Prism 5.

## Results

### Stromal Cells and Leukocytes are Clustered in Lymphoid-like Structures in Aneurysmal Aortas

**Figure 1:**
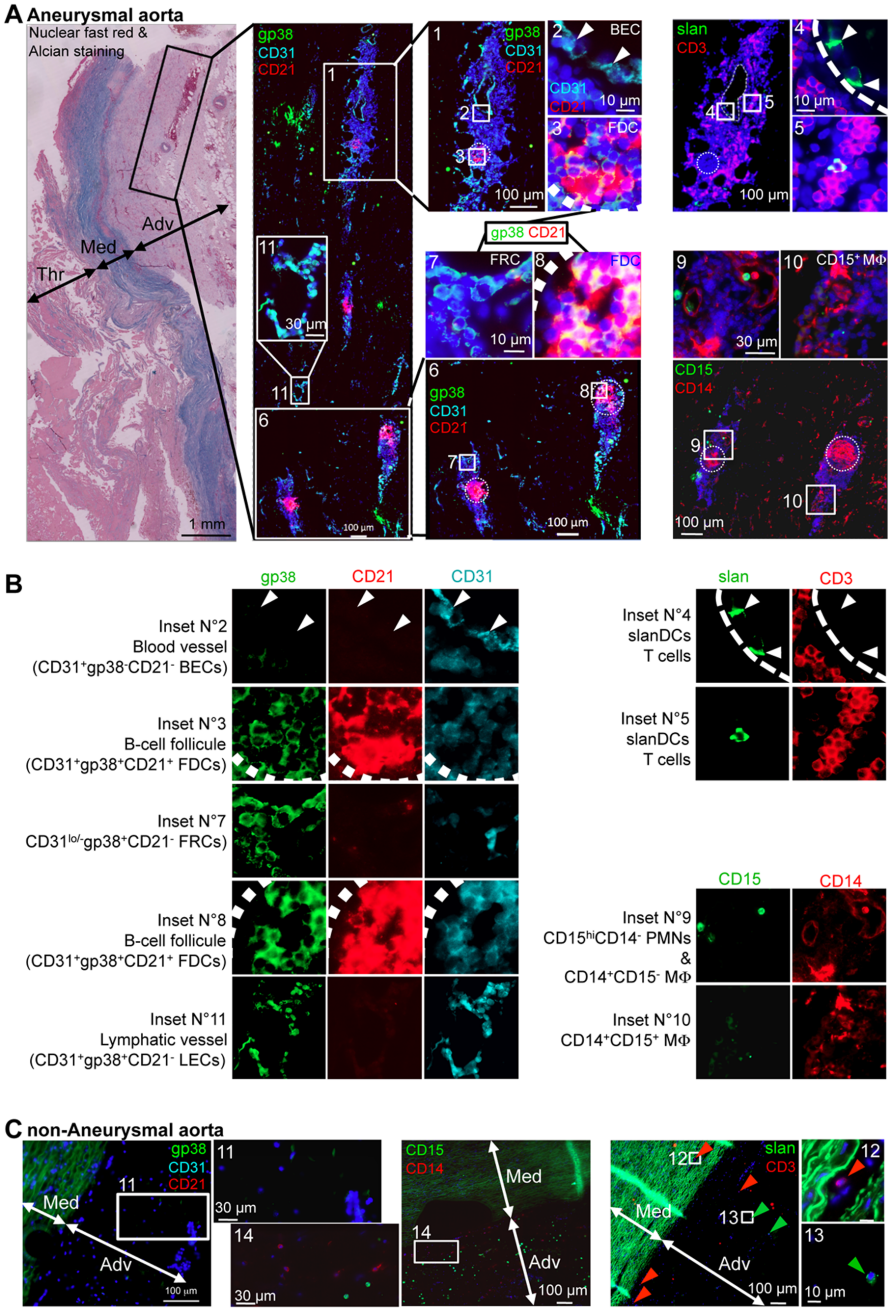
Blood and lymphatic vessels are found in the adventitia of aneurysmal aortas, which also comprises lymphoid structures composed of stromal cells and leukocytes. Reresentative immunohistofluorescence analyses of paraffin-embedded (**A**) aneurysmal or (**B**) non-aneurysmal aortas. (**A**) The far left panels shows a micrograph of a nuclear fast red and Alcian blue-stained section. The left immunofluorescent cross-section micrograph corresponds to gp38 (green), CD31 (cyan) and CD21 (red) staining to study stromal cell subsets localization, and corresponds to the region defined by a black rectangle in the Nuclear fast red & Alcian micrograph. Magnifications of regions defined by white rectangles are shown as insets and in the two middle panels, numbered white boxes indicate the position of the magnified insets, and cell subsets defined in insets are sometimes specified. In each middle panel inset, two out of the three staining (CD31 and CD21 in the upper inset, or gp38 and CD21 for the other insets) are shown. Sequential sections following the one stained for gp38/CD31/CD21 were stained for slan (M-DC8, green) and CD3 (red) to detect slanDC and CD3^+^ T cells (upper right panels), or for CD15 (green) and CD14 (red) to detect macrophages (CD14^+^CD15^+/−^) and polymorphonuclear cells (CD15^+^CD14^−^) (lower right panels). Numbered white boxes indicate the position of the magnified insets. (**B**) Single channels for insets defined in (**A**) are show. The cell types and/or structure defined are written on the left of each inset. (**C**) Sequential sections of a paraffin embedded non-aneurysmal aorta were stained for gp38 (green), CD31 (cyan) and CD21 (red) (left panels), for slan (M-DC8, green) and CD3 (red) (middle panels), or for CD15 (green) and CD14 (red) (right panels). Numbered white boxes indicate the position of the magnified insets. Thr, thrombus; Med, media; Adv, adventitia.

The presence of Arterial Tertiary Lymphoid organs (ATLOs) has been detected in the adventitia from aneurysmal aortas of a large number of patients in our laboratory. Here, we carried out immunofluorescent analyses on arterial cross-sections (Figure 1) to evaluate the topology of and composition in stromal cell and leukocyte subsets of the adventitia. Images obtained using serial cross-sections from representative aneurysmal (Figure 1A and 1B) and non-aneurysmal aortas are shown (Figure 1C).

For aneurysmal aortas (Figure 1A and 1B), we focused our attention on regions of the adventitia, in regards of the thrombus, presenting cellular clusters (black box in the Nuclear fast red & Alcian histochemical staining, Figure 1A). Single channels of insets are displayed in Figure 1B.

Large cellular clusters (inset N°1) were often crossed by blood vessels covered by CD31^+^gp38^−^CD21^−^ Blood endothelial cells (BECs, inset N°2: CD31 and CD21 channels displayed but not the gp38 channel). We also detected vascular structures that likely correspond to lymphatic vessels, since they were covered by CD31^+^gp38^+^CD21^lo^ Lymphatic Endothelial Cells (LECs) (inset N°11). Large cellular clusters (inset N°1) but also smaller cellular clusters (inset N°6) comprised also CD21-positive zones corresponding to Follicular Dendritic Cells (FDCs) that are known to cluster in B-cell zones (germinal centers delineated by circle dotted lines) of secondary lymphoid organs (SLO). Magnifications of FDC-rich zones are shown in insets N°3 and N°8. CD21^+^ FDCs were also positive for gp38 as observed in insets N°3 and N°8, but also for CD31. In the periphery of these lymphoid structures, clusters of cells positive for gp38, weakly expressing CD31 (as compared to BECs) but negative for CD21 (inset N°7) were probably corresponding to clusters of CD31^−^gp38^+^CD21^−^ Fibroblastic Reticular Cells (FRCs).

slan/M-DC8 (slanDC) and CD3 (T lymphocytes) staining were done simultaneously on serial cross-sections (Figure 1A and 1B, upper right panels). Interestingly, we observed that slanDCs were either interacting with the blood vessel endothelium (as illustrated by white arrow heads on both insets N°2 & 4), or in close proximity to CD3^+^ T cells (inset N°5). CD3^+^ T cells were not found in this “CD21-positive B-cell zone” (circle dotted line) but rather surrounding it, as observed in SLOs. Thus, these lymphoid-like clusters strongly resemble SLOs and correspond to the previously described arterial tertiary lymphoid organs (ATLOs).

On an additional serial cross-section, we have also evaluated the localization of macrophages (CD14^+^CD15^+/−^) and polymorphonuclear cells (PMNs, CD14^−^CD15^hi^) (Figure 1A and 1B, lower right panels). CD14 and CD15 double staining of the region containing the two small ATLOs magnified in inset N°6 is shown. First, we confirmed that like macrophages, stromal FDCs are strongly positive for CD14 [25] since cells in the same regions (circle dotted lines) as CD21^+^ FDC (inset N°6) are strongly positive for CD14 (CD15/CD14 image). Only few cells were strongly CD15-positive and showed no CD14 expression thus corresponding to PMNs (inset N°9). Contrary to FRCs, FDCs, T cells and slanDC that were detected almost only in ATLOs, CD14^+^CD15^−^ cells with a macrophage morphology were distributed homogeneously in the adventitia but were also eventually found in clusters in ATLOs (inset N°10). Interestingly, contrary to macrophages found in non-ATLO regions, these clustered macrophages in ATLOs were also weakly positive for CD15 thus corresponding to the CD15^+^ “M1-like” macrophages (inset N°10).

The same staining strategy was carried out on serial cross-sections of non-aneurysmal aortas (Figure 1C). Elastic fibers of the media were strongly autofluorescent and are depicted in green. No ATLO-like cellular clusters, nor blood or lymphatic vessel could be detected and the density of nucleated cells in the adventitia was much lower as compared to that observed in the aneurysmal adventitia. In the gp38/CD31/CD21 staining, only weakly gp38-stained CD21^−^CD31^−^ fibroblasts could be detected (inset N°12). In the CD15/CD14 staining, macrophages and PMNs were found all over the adventitia (inset N°15), these later being more densely detected in the adventitia from non-aneurysmal aortas (Figure 1C) as compared to the aneurysmal aortas (Figure 1A, lower right panels). Finally, in the slan/CD3 staining, dispersed slanDCs (green arrow heads and inset N°13) and CD3^+^ T cells (red arrow heads) cells could be detected in the adventitia. Interestingly, we were able to detect CD3-positive T cells in the media (inset N°12).

### Comparative Analysis of Leukocyte Subsets in the Media and the Adventitia of Aneurysmal and Non-aneurysmal Aortas

**Figure 2:**
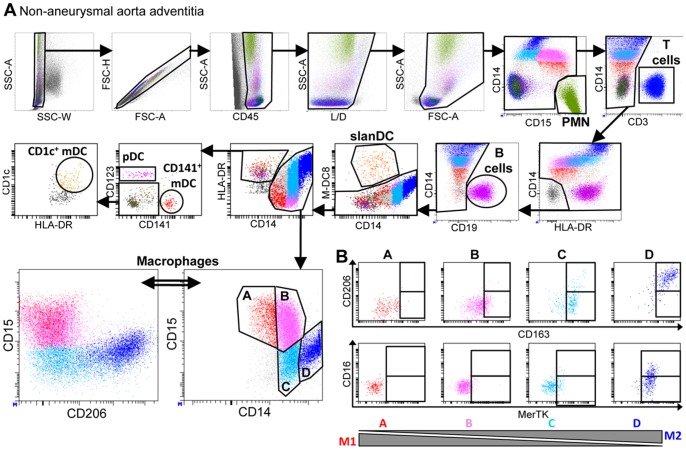
Analysis of leukocyte subsets in the adventitia from a non-aneurysmal abdominal aorta by polychromatic flow cytometry. (**A**) The gating strategy to analyze CD45^+^ leukocytes from a non-aneurysmal abdominal aorta by 15-color flow cytometry is shown. This strategy allows the simultaneous definition of CD14^−^CD15^hi^ polymorphonuclear cells (PMN), CD3^+^ T cells, CD19^+^ B cells, CD14^+^ macrophages, and dendritic cell DC) subsets comprising CD14^lo^M-DC8^+^ slanDC, and among lin^−^HLA-DR^+^ events, CD123^+^ plasmacytoid DC (pDC), and two subsets of myeloid DC (mDC), CD141^+^ mDC and CD1c^+^ mDC. The lower right dot plot shows four macrophage subsets defined by their relative expression of CD14 and CD15, and the lower left panel shows CD206 and CD15 expression by these macrophage subsets. (**B**) Flow cytometric dot plots of CD163 and CD206 (upper dot plots) and MerTK and CD16 (lower dot plots) expression by the four macrophage subsets defined in (**A**).

Following the detection using immunohistofluorescence of multiple cellular subsets of both stromal and hematopoietic origin that (apart from macrophages), clustered in adventitial ATLOs, we next wanted to define quantitatively and more precisely these subsets by flow cytometry. Flow cytometry not only provides a quantitative analysis of these cell subsets but also allows studying more cell subsets (such as dendritic cell subsets) that sometimes require more than 4 markers to be defined. Thus, in order to study all leukocyte subsets simultaneously in the media or in the adventitia, we carried out 15-color flow cytometric analyses (Figure 2). To manage spectral overlapping of fluorochromes used here, antibodies coupled to fluorochromes that strongly overlapped were directed against antigens that were not co-expressed. For example, all Brilliant Violet coupled antibodies were directed against lineage markers that are by definition not co-expressed (Table S1). Also, fluorochromes such as V500 that have a low brilliance intensity were associated to strongly expressed molecules such as CD45 for instance.

Total cells extracted by mechanic disruption and enzymatic digestion of medial and adventitial tissues from non-aneurysmal and aneurysmal aortas were studied (Table 1). The gating strategy is shown for a representative non-aneurysmal abdominal aorta adventitia (Figure 2A). In these analyses, among singlet (SSC-W/SSC-A and FSC-H/FSC-A dot plots) CD45^+^ live (Live/Dead^−^) cells, CD15^hi^CD14^−^polymorphonuclear cells (PMN, green), CD3^+^ T cells (blue), CD19^+^ B cells (purple), CD14^lo^M-DC8^+^ slanDC (orange), CD14^+^ macrophages, and among lin^−^HLA-DR^+^ cells, CD123^+^ plasmacytoid dendritic cells (pDC, pink), CD141^+^ (BDCA-3) myeloid DC (mDC, red) and CD1c^+^ (BDCA-1) mDC (beige) were simultaneously defined. Macrophages were further subdivided in four subsets based on the relative expression level of CD14 and CD15, CD15^+^CD14^lo^ (subset A, red), CD15^+^CD14^int^ (subset B, pink), CD15^−^CD14^int^ (subset C, cyan), and CD15^−^CD14^hi^ cells (subset D, blue). The relative expression of CD15 and of the mannose receptor (CD206) by these four subsets is shown, demonstrating that CD206 is strongly expressed only by CD15^−^CD14^hi^ (subset D, blue) macrophages. CD206 being described as expressed, at least *in vitro*, by M2-polarized macrophages, we further investigated the expression of other M2-associated molecules, CD163, CD16 and MerTK, the later being described as expressed by M2c-polarized macrophages (Figure 2B) [17], [26], [27]. While CD15^+^CD14^lo^ cells (subset A) showed no expression of these molecules, CD15^−^CD14^hi^ cells (subset D) were strongly positive for CD163, CD206, MerTK, and partially positive for CD16. The CD15^+^CD14^int^ (subset B) and CD15^−^CD14^int^ (subset C) subsets had intermediate phenotypes, with progressive acquisition of the molecules. We thus defined CD15^+^CD14^lo^ macrophages (subset A) as M1-like cells, CD15^−^CD14^hi^ macrophages (subset D) as M2-like cells and the two other subsets as intermediate cells between these two polarization states.

**Figure 3:**
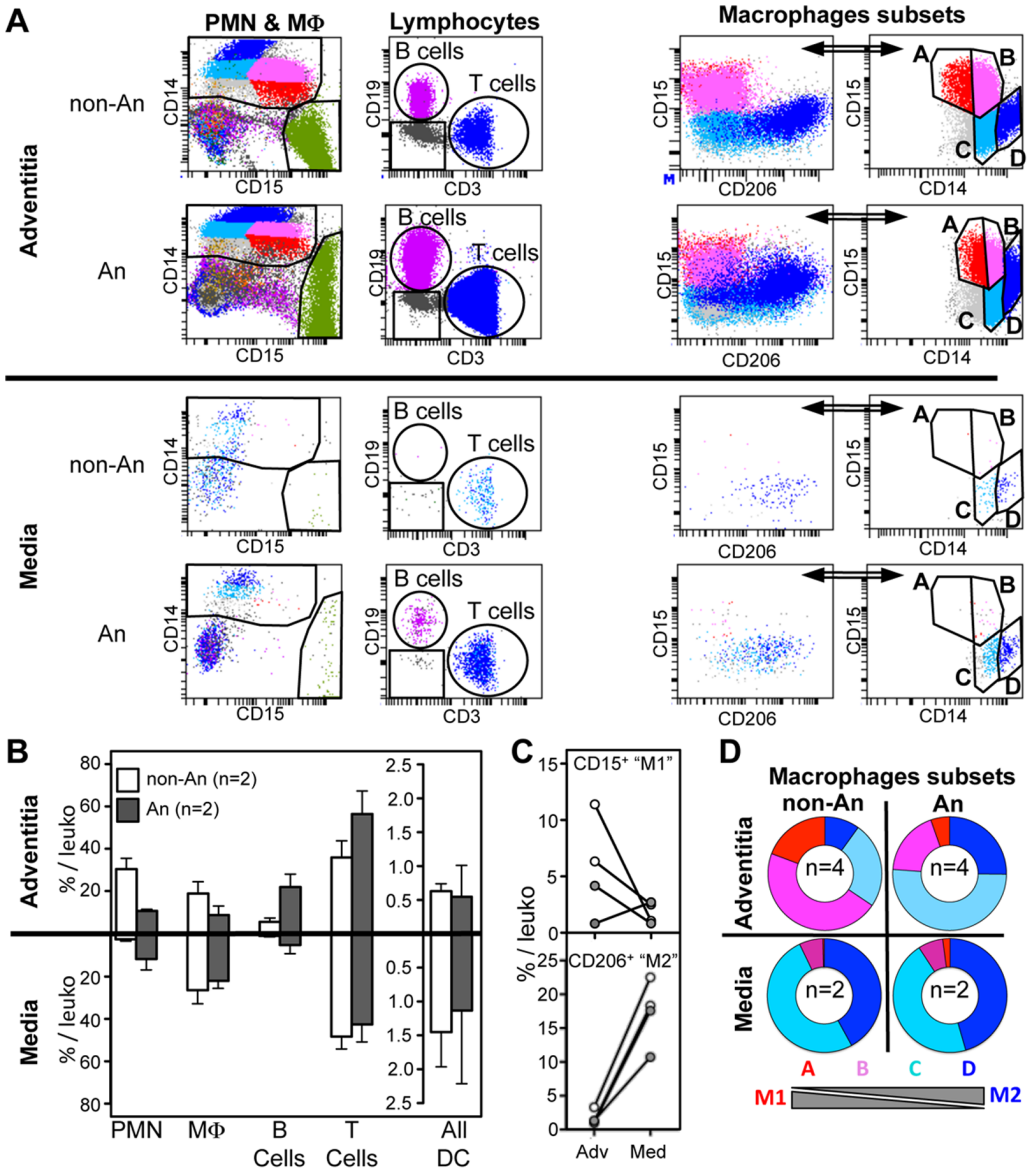
Comparison of leukocyte subsets in the adventitia and the media from non-aneurysmal and aneurysmal abdominal aortas. (**A**) Flow cytometric dot plots defining polymorphonuclear cells (PMN) and macrophages (MΦ, far left dot plots), CD3^+^ T cells and CD19^+^ B cells (middle left dot plots), and macrophages subsets (right dot plots) in the adventitia (upper panels) and the media (lower panels) from a non-aneurysmal (non-An) and an aneurysmal (An) abdominal aorta. (**B**) On the left, graphical representation of the percentages among total CD45^+^ leukocytes of PMN, macrophages, B cells, T cells and all DC in the adventitia (n = 2, upper histograms), and the media (n = 2, lower histograms) from non-An (white histograms) and An (grey histograms) aortas. (**C**) Comparison between the adventitia (Adv) and the media (Med) for the percentages among total CD45^+^ leukocytes of CD15^+^ and CD206^+^ macrophage subsets in non-An (white filled circles) and An (grey filled circles) aortas (lines associates paired results obtained in the adventitia and the media from the same patient). (**D**) Ring graphical representation of the proportion of the macrophage subsets defined in Figure 2A among total macrophages in the adventitia (n = 4, top panels) and the media (n = 2, bottom panels).

Total cell suspensions from the adventitia and the media of four abdominal aortas [two non-aneurysmal (non-An) and two aneurysmal (An)] were compared for leukocyte content (Figure 3). Flow cytometric dot plots show PMN, B and T lymphocytes, and macrophages (M Φ) subsets detected in the adventitia and the media from one non-aneurysmal and one aneurysmal abdominal aorta (Figure 3A). While all major leukocyte subsets could be detected in the adventitia from abdominal aortas, non-aneurysmal medias leukocytes were mostly composed of macrophages, dendritic cells (whose proportion tended to be increased as compared to adventitia) and T cells, but almost no B cells and PMN could be detected (Figure 3A and 3B). This is in line with the detection of CD3^+^ T cells in the media from non-aneurysmal aortas in our immunohistofluorescent experiments (Figure 1C, inset N°12). Interestingly, in contrast to the media from the two non-aneurysmal abdominal aortas, PMN could be detected in the two aneurysmal medias. It was also the case for B cells, which might indicate that ATLO can develop within the media in certain circumstances. In the media from both non-aneurysmal and aneurysmal abdominal aortas, macrophages were nearly all M2-like since they strongly co-expressed CD14 and CD206, while they did not express CD15 (Figure 3A). Indeed, while the percentage among total leukocytes of CD15^+^ “M1-like” macrophages dropped in the media as compared to the adventitia from 3/4 aortas (both aneurysmal and non-aneurysmal), it was the contrary for CD206^+^ “M2-like” macrophages in the four abdominal aortas tested (Figure 3C). Also, as compared to non-aneurysmal abdominal aortas (n = 4), the proportion of macrophages subsets was deeply modified in the adventitia of aneurysmal abdominal aortas (n = 4) with a strong decrease of M1-like macrophages in favor of M2-like macrophages (Figure 3D).

**Figure 4:**
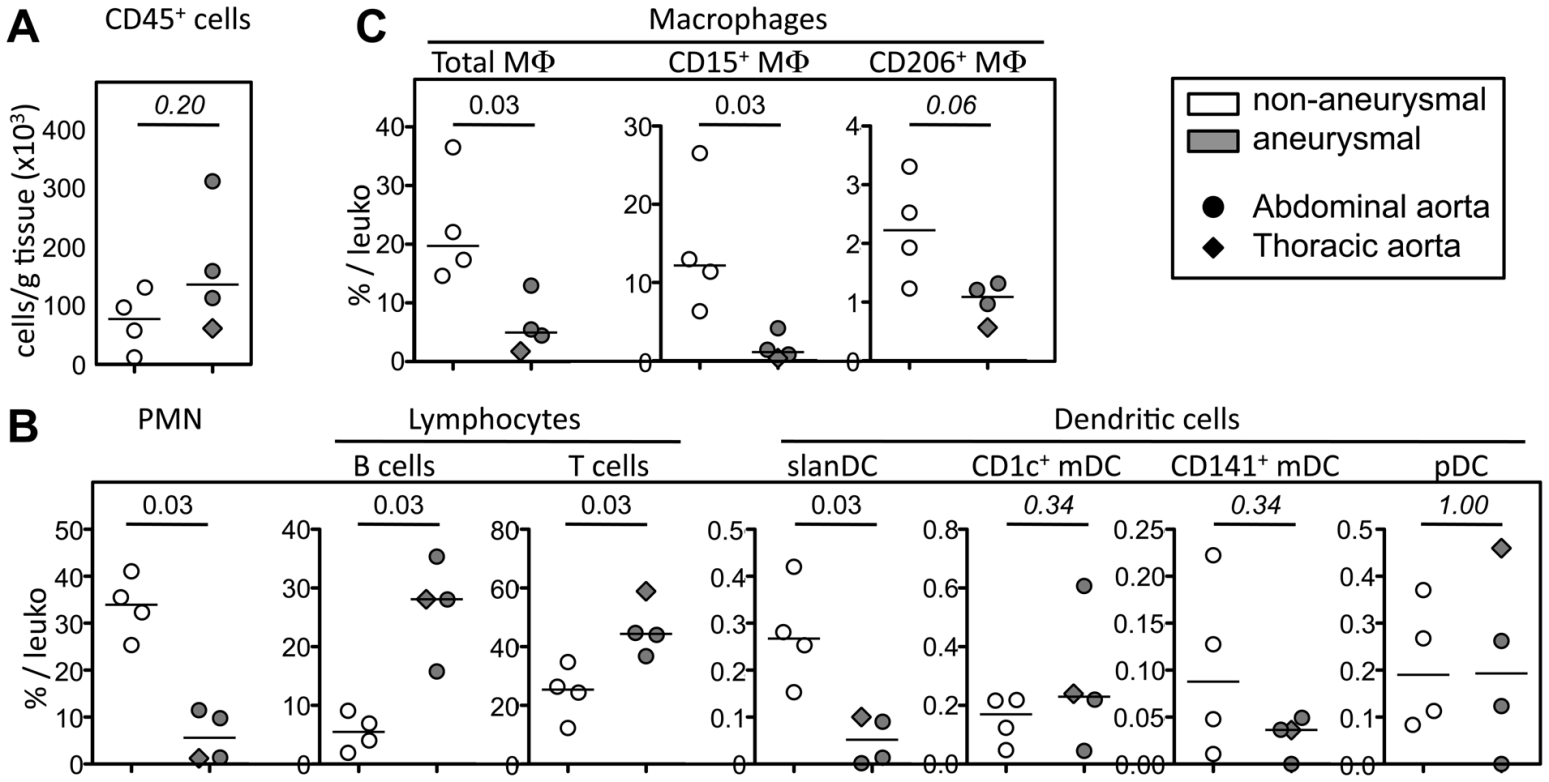
Proportion of leukocyte subsets in the adventitia of non-aneurysmal and aneurysmal aortas. (**A**) Graphical representation of the density of CD45^+^ leukocytes (cells/g tissue), and of (**B,C**) the percentage among CD45^+^ leukocytes of (**B**) PMN, lymphocyte, DC, and (**C**) macrophages and their subsets in the adventitia from non-aneurysmal (white filled symbols, n = 4) and aneurysmal (grey filled symbols, n = 4) aortas. Circles correspond to abdominal aortas, and the diamond to a thoracic aorta. p values were calculated with the Mann-Whitney nonparametric test.

We next compared the proportion and the density (cells/g tissue) of all leukocyte and macrophage subsets in aortic adventitia (Figure 4 and Figure S1). Although the density of CD45^+^ leukocytes trended to be increased in aneurysmal aortas, this was not significant (p = 0.20, Figure 4A). Concerning leukocyte subsets, while B cell (p = 0.03) and T cell (p = 0.03) proportions were significantly increased in aneurysmal aortas, most probably linked to the their accumulation in ATLOs (see Figure 1A), it was the contrary for PMN (p = 0.03), slanDC (p = 0.03) and total CD14^+^ macrophages (p = 0.03) whose proportions significantly decreased (Figure 4B and 4C). Among macrophages subsets, only CD15^+^ “M1-like” macrophages proportion among total leukocytes were significantly decreased in aneurysmal aortas (p = 0.03), most probably driving the decrease of the proportion of total macrophages (Figure 4C).

### Comparative Analysis of Stromal Cell Subsets in Aneurysmal and Non-aneurysmal Aortas

**Figure 5:**
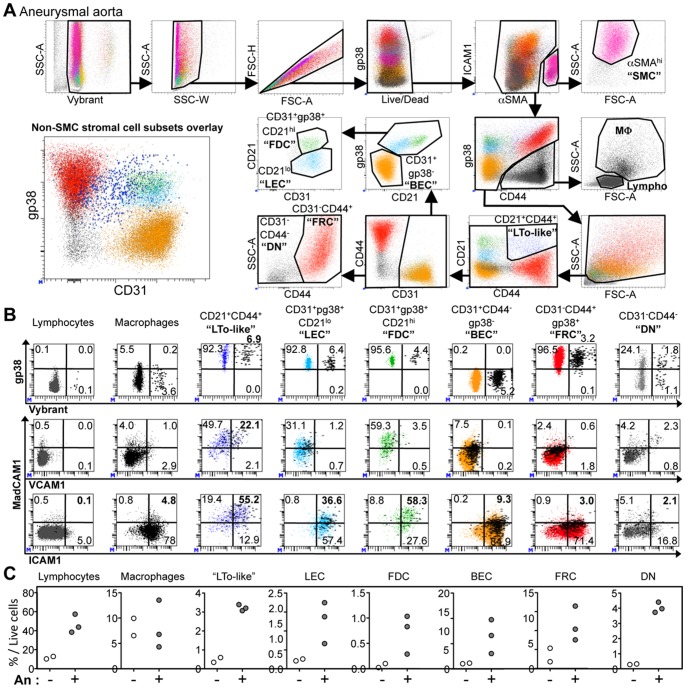
Analysis of stromal cell subsets in the adventitia form non-aneurysmal and aneurysmal aortas. (**A**) The gating strategy to analyze all nucleated cells (Vybrant^+^) and particularly stromal cell subsets from an aneurysmal artery by 10-color flow cytometry is shown. This strategy allows the simultaneous definition of residual aSMA^hi^ smooth muscle cells (SMC), CD44^+^gp38^lo/−^ leukocytes [macrophages (MΦ) and lymphocytes (Lympho) defined on the forward (FSC-A) and side (SSC-A) scatter], among non-leukocyte cells, CD21^+^CD44^+^ “LTo-like” cells, CD31^+^CD44^−^pg38^−^ blood endothelial cells (BEC), CD31^+^gp38^+^CD21^lo^ lymphatic endothelial cells (LEC), CD31^+^gp38^+^CD21^hi^ follicular dendritic cells (FDC), CD31^−^CD44^+^ fibroblastic reticular cells (FRC), and CD31^−^CD44^−^ double negative cells (DN). The lower left dot plot (CD31 vs pg38) shows the overlay of all stromal cell subsets excluding SMC. (**B**) Flow cytometric dot plots showing the expression of Vybrant (Vybrant^hi^ cells being undergoing division) and gp38 (upper panels), VCAM1 and MadCAM1 (middle panels) or ICAM1 and MadCAM1 (lower panels) for cell subsets defined in (**A**). The percentage of cells in each quadrant is indicated. Vybrant-positive events are highlighted and displayed as thick black dots in the middle and lower VCAM1/ICAM1 vs MadCAM1 dot plots. (**C**) Comparison of the percentage among total live cells of cell subsets defined in (**A**) in the adventitia from two non-aneurysmal (white filled symbols) and three aneurysmal (white filled symbols) abdominal aortas.

The composition in stromal cells in the adventitia of human aorta has never been defined precisely. Our immunofluorescent experiments pointed to a greater density and diversity of stromal cells in the adventitia from aneurysmal aortas as compared to non-aneurysmal aortas. To define stromal cells quantitatively and more precisely, we carried out 10-color flow cytometric analyses on total cells extracted by mechanic disruption and enzymatic digestion of two non-aneurysmal abdominal aortas and three aneurysmal aortas (Figure 5 and Figure S2). The gating strategy is shown for an aneurysmal aorta (Figure 5A) since only very few stromal cells could be recovered from non-aneurysmal aortas (Figure S2A).

In these analyses, to distinguish cellular events from debris linked to the mechanical and enzymatic cellular isolation process, the Vybrant fluorescent DNA selective-stain was used to define nucleated cells and to evaluate the proportion of cells undergoing mitosis. This had not been necessary for the analysis of leukocytes since they could all be distinguished from debris by their strong CD45 expression. Thus, following a first step of nucleated cell selection (Vybrant^+^), among singlet (SSC-W/SSC-A and FCS-H/FSC-A dot plots), live (Live/Dead^−^) cells, residual αSMA^hi^ gp38^−^ smooth muscle cells (SMC, pink) were excluded. Next, CD44^+^gp38^lo/−^ cells corresponding to leukocytes were separated from stromal cells that comprised the following subsets: CD21^+^CD44^+^ cells that we called “LTo-like” linked to their phenotype (see Figure 5B, LTo for Lymphoid Tissue Organizer, blue), CD31^+^gp38^−^ blood endothelial cells (BEC, beige), CD31^+^gp38^+^CD21^lo^ lymphatic endothelial cells (LEC, cyan), CD31^+^gp38^+^CD21^hi^ follicular dendritic cells (FDC, green), CD31^−^CD44^+^gp38^+^ fibroblastic reticular cells (FRC, red) and CD31^−^CD44^−^gp38^−^ double negative cells (DN, grey).

The *in vivo* proliferation (evaluated by the proportion of Vybrant^hi^ cells), and the expression of gp38, MadCAM1, ICAM1 and VCAM1, four molecules known to be expressed by LTo cells, were evaluated for lymphocytes, macrophages and the different stromal cell subsets (Figure 5B and Figure S2). Surprisingly, lymphocytes showed an almost total absence of proliferating cells (0.1%) while macrophages weakly proliferated (3.8%). Interestingly, macrophages from aneurysmal aortas showed a greater proliferation index as compared to non-aneurysmal aortas (Vybrant^hi^: 1.2 and 1.3% in the two non-aneurysmal adventitia; 3.8, 4.4 and 4.6% in the three aneurysmal adventitia), which was not the case for all the other cellular subsets (Figure S2). In both non-aneurysmal and aneurysmal adventitia, all stromal cell subsets proliferated, particularly CD21^+^CD44^+^ LTo-like cells (6.9% proliferating cells, Figure 5B). These later strongly expressed MadCAM1 and ICAM1, and were also the only cells that expressed VCAM1 (22.1%). Indeed, those expressing VCAM1 were proliferating (Vybrant^hi^ proliferating cells being represented as black thick dots reported on the VCAM1/MadCAM1/and ICAM1/MadCAM1 dot plots). Therefore, the co-expression of gp38, MadCAM1, ICAM1 and VCAM1 by these proliferating CD21^+^CD44^+^ cells further confirm that they are LTo-like cells.

Next, we compared the proportions of leukocyte and stromal cells in non-aneurysmal and aneurysmal aortas (Figure 5C). As expected, we confirmed here that the proportion of lymphocytes increased. Interestingly, proportions of LTo-like cells, LEC, FDC, BEC, FRC and DN stromal cell subsets were greater in the adventitia of aneurysmal aortas as compared to non-aneurysmal aortas (Figure 4D).

## Discussion

Atherothrombosis represents a complex remodeling of arteries that affects all their layers. Events taking place in the intima received a particular attention since thrombosis occurs at this interface. Destruction of the media has been also extensively studied, especially in the context of aneurysms. Only recently, cellular and molecular events taking place in the adventitia have started to be deciphered. Indeed, the adventitia of atherothrombotic vessels is deeply reshaped, supports an intense angiogenesis [28] and can be infiltrated by leukocytes. The “outside-in” hypothesis [29] states that vascular neoangiogenesis and its associated inflammation is initiated in the adventitia and progresses towards the intima where it would promote plaque complication (intraplaque hemorrhage and/or plaque rupture) [1], [30], [31].

In the present study, we described the cellular network in the adventitia from aneurysmal aortas and compared it to that of non-aneurysmal aortas. This was done by combining 4-color immunohistofluorescence, that provided topographical information, and by multicolor flow cytometry that provided quantitative and more qualitative information on stromal and leukocyte subsets comprised in human aortic non-aneurysmal and aneurysmal adventitia. Our study revealed that the adventitia from both aneurysmal and non-aneurysmal aortas contains a much greater diversity of cells of both hematopoietic and non-hematopoietic (stromal) origin, than commonly thought. Although non-aneurysmal organ donors were younger than aneurysmal patients (median age: non-aneurysmal, 47; aneurysmal, 65; see Table 1), which is an inevitable bias since organ donors are selected among young candidates while aneurysm develops mostly in older individuals, this likely does not explain differences between non-aneurysmal and aneurysmal aortas since no correlation could be observed between the age and proportions of cell subsets in this study (data not shown). Also, all tissue samples were collected in similar conditions since non-aneurysmal tissues were from brain dead organ donors that still had a beating heart, and aneurysmal tissues were from surgical interventions on living patients.

Concerning hematopoietic cells, we confirmed that aneurysmal aortas contain greater proportions of B and T cells as compared to non-aneurysmal aortas. It was the contrary for macrophages, slanDCs and polymorphonuclear cells (PMN), these later being defined as large (FSC-A^hi^), granular (SSC-A^hi^) CD15^+^HLA-DR^−^ cells. This phenotypic definition of PMN could include some mast cells but unpublished results from our laboratory indicate that these later are rare in the adventitia from both non-aneurysmal and aneurysmal aortas (less than 50 mast cells/g of aortic adventitia, while we detected an average of 19.000 PMN/g of aortic adventitia, Figure S1).

As previously described [7], ATLOs were in close proximity to blood vessels (Figure 1A, inset 1) indicating that they receive a blood supply from where naïve lymphocytes could be recruited. Macrophages and stromal cells had substantial proportions of Vybrant^hi^ proliferating cells. Interestingly, while stromal cell subsets showed similar proliferation in the adventitia from aneurysmal and non-aneurysmal aortas, macrophages showed a greater percentage of proliferation in aneurysmal aortas. This is in line with the recent report from Robbins *et al.* showing that local proliferation, rather than de novo recruitment, dominates lesional macrophage accumulation in atherosclerosis [32]. Alternatively, macrophages could have been recruited from inflamed vasa vasorum vessels in the adventitia and could have then spread away and migrated all over the adventitia. Furthermore, unlike lymphocytes, macrophages were not detected only in close proximity to blood vessels but were homogeneously distributed in the entire adventitial layer (Figure 1A, CD14/CD15 staining), both in aneurysmal and non-aneurysmal aortas, suggesting that the adventitial macrophage contingent originates from resident macrophages rather than from recruited monocytes.

Contrary to Boytard *et al.*, who described a predominance of pro-inflammatory CD206/(Mannose Receptor)-negative macrophages in the adventitia adjacent to aneurysm [33], we found an increased proportion of M2-like CD206^+^ macrophages in the adventitia from aneurysmal as compared to non-aneurysmal aortas. Adventitial CD206^+^ macrophages might be implicated to clear molecules radially convected from the intima. Indeed, owing to the pressure generated, mechanical stress/strain is generated within the arterial wall, and radial hydraulic conductance occurs across the wall, conveying soluble substances from the blood outwards [34]. In the context of abdominal aortic aneurysm (AAA), where erythrocytes and platelets are massively accumulated in the luminal thrombus, adventitial macrophages could endocytose molecules derived from these cells. In accordance, it has been recently demonstrated that hemoglobin-derived iron endoctysosis and intracellular retention, induced and M2 differentiation process of human macrophages *in vitro* [35]. This increased proportion of M2-like CD206^+^ macrophages in aneurysmal adventitia might also be imposed by the requirement for tissue repair, a function for which M2-macrophages are particularly competent [15], [17].

The proportion of M1-like CD206^−^CD15^+^ macrophages, known to produce TNFα, was reduced in aneurysmal adventitia but they were detected in clusters at the edge of ALTOs as observed in immunofluorecence experiments. TNFα, that participates in adventitial inflammation, could arise from these CD206^−^CD15^+^ M1-like macrophages or from other cells such as slanDCs, which are strong TNFα producers in response to the microbial product lipopolysaccharide (LPS), that was previously detected in aneurysmal tissues [36]. SlanDCs from chronically HIV-1 infected patients secrete much greater amounts of TNFα than slanDCs from non-infected patients indicating that in a chronic inflammatory situation, even very small number of these pro-inflammatory cells can lead to great amounts of TNFα production [19]. We also observed that slanDCs were present in substantial proportion in the adventitia from non-aneurysmal aortas. During the first stages of aneurysm development, via their capacity to produce high amounts of TNFα, these cells could play the role of “lymphoid tissue inducer” (LTi) cells by stimulating stromal “lymphoid tissue organizer” LTo cells, thus initiating ATLO formation. Also, slanDCs strongly express the chemokine receptor CX3CR1 and migrate in response to its ligand, CX3CL1. This chemokine can be found in a soluble chemo-attracting form, or bound at the cell membrane of vascular endothelial cells following their stimulation by pro-inflammatory signals, thus favoring CX3CR1^+^ cells adhesion and extravasation from the blood to inflamed tissues [37]. CX3CL1, which has a major pathogenic role during atherosclerosis [38], [39], was detected in aneurysmal adventitia where it is produced by vascular endothelial cells such as BECs in response to TNFα [40]. Of note, we have detected slanDCs that were interacting with BECs within an ATLO (Figure 1A, inset 4), an interaction possibly mediated by CX3CL1 bound on these endothelial cells. Therefore, a vicious circle involving slanDCs, bacterial LPS, TNFα and CX3CL1 could maintain the chronic inflammation that is associated to aneurysm progression, where slanDCs would favor TNFα-mediated CX3CL1 production, which in turn would promote slanDC and other CX3CR1^+^ inflammatory cell recruitment, all together leading to both ATLO formation and aneurysm progression.

The precise nature of slanDCs is not known so far, they may be a subset of non-classical CD14^lo^CD16^hi^ monocytes [41] or pro-inflammatory DCs [42]. In both cases, in addition to their pro-inflammatory function, they could act as suitable antigen presenting cells since they were shown to efficiently present antigens to autologous T lymphocytes [18], and a recent work indicates that in mice, monocytes can be found in tissues where they acquire antigens that they transport to lymph node, a function redundant with DCs [43]. Therefore, our observation of a slanDC in a T-cell zone of an ATLO (Figure 1A, inset 5) indicates that these cells could also present antigens to T cells and thus participate to the induction of T cell responses in ATLOs. This is in line with the recent observation of slanDCs in lymphoid follicle-like structures in the lesional skin of Lupus erythematosus patients [44].

Concerning stromal cells, it is commonly thought that adventitia is only composed of fibroblasts and blood endothelial cells (BECs) [29]. To our knowledge, this study is the first to comprehensively define both qualitatively and quantitatively the aortic adventitial stromal cell network. We could observe that an important diversity of stromal cells can be detected in both non-aneurysmal and aneurysmal adventitia, and that they were much more abundant in aneurysmal adventitia.

Importantly, unlike non-aneurysmal adventitia, cells phenotypically resembling to lymphatic endothelial cells (LECs) were detected in aneurysmal adventitia by flow cytometry and vessels layered by gp38^+^CD31^+^CD21^lo^ endothelial cells were detected by immunohistochemistry (Figure 1A and 1B, inset N°11) indicating that lymphangiogenesis is taking place in the adventitia of aneurysmal aortas.

In aneurysmal adventitia, we also detected cells phenotypically resembling follicular dendritic cells (FDC) that are crucial to maintain and activate B cells within germinal centers in secondary lymphoid organs. Contrary to mouse FDCs [45], CD21^hi^ “FDC-like” cells that we detected by flow cytometry expressed CD31. This expression was confirmed in our immunofluorescent analyses on tissue cross-sections since gp38, CD21 (hallmark of FDCs), and CD31 co-localized in FDC-rich zones in ATLOs (Figure 1B, insets N°3 and N°8), thus confirming that FDCs were positive for CD31.

The nature of LTo cells that induce the formation and maintain ATLOs in the context of mouse atherosclerotic aortas was hypothesized to be SMCs [46], but in our flow cytometric experiments, αSMA^hi^ SMC, which probably correspond to contamination by media, were negative for gp38, VCAM1 and expressed only low levels of ICAM1, all markers of LTo cells. Interestingly, we were able to identify a subset of CD21^+^CD44^+^ cells that we called “LTo-like”, since they were the only stromal cell subset to co-express gp38, VCAM1 and ICAM1.

Finally, the implication of myofibroblasts in the progression of various cardiovascular diseases such as thoracic aortic aneurysm (TAA) has been suggested [47]. Although such cells were detected in the adventitia of inflammatory aneurysmal aortic tissue cross-sections based on morphologic and αSMA staining [48], we did not detect myofibroblast-like cells (gp38^+^αSMA^hi^) in any of the aneurysmal aortas that we have studied.

In summary, we have precisely described the cellular composition of human arterial adventitia, both at steady state and in the setting of aneurysms. First, this allowed us to show that unlike the medial layer (at least at steady state) that besides SMCs comprises mostly macrophages and T cells, the adventitia comprises a much greater variety of leukocytes. Second, we observed an altered balance in macrophages subsets in favor of M2-like macrophages, an increased proliferation of macrophages, and a greater number of all stromal cells in aneurysmal aortas. Third, we also confirmed that in this pathological setting, blood vessels and ATLOs were detected in the adventitia, but we showed for the first time that these lymphoid structures comprised also slanDCs that could participate in the induction of T-cell responses. Fourth, we showed that lymphatic vessels can be detected in aneurysmal adventitia, the function of which will have to be evaluated in future studies. It will be of particular interest to determine whether they are connected to afferent and/or efferent lymphatics. This will indicate whether they connect ATLOs to the systemic immune system and indirectly, this will indicate whether immune responses taking place in ATLOs are exclusive or not to the adventitia.

## Supporting Information

Figure S1 **Density of leukocyte and stromal cell subsets in the adventitia of aneurysmal and non-aneurysmal aortas.** Graphical representation of the density (cells/g tissue) of (A) PMN, lymphocyte, DC, (B) macrophage, and (C) stromal cell subsets in the adventitia from non-aneurysmal (white filled symbols, n = 5) and aneurysmal (grey filled symbols, n = 5) aortas. Circles correspond to abdominal aortas, and diamond to a thoracic aorta. p values were calculated with the Mann-Whitney nonparametric test.
(PDF)

Figure S2 **Comparison of the phenotype and the proliferation of all cell subsets in the adventitia from non-aneurysmal and aneurysmal aortas.** (A) The gating strategy to analyze all nucleated cells (Vybrant^+^) and particularly stromal cell subsets from a non-aneurysmal aorta by 10-color flow cytometry is shown. (B) Comparison of the percentage among the parent population of proliferating Vybrant^hi^ cells for cell subsets defined in (Fig. 5A) in the adventitia from two non-aneurysmal (white filled symbols) and three aneurysmal (grey filled symbols) aortas.
(DOC)

Table S1 Configuration of the BD FACS LSRII used in the present study.
(PDF)
