# Supplementary material for: Deciphering the Stromal and Hematopoietic Cell Network of the Adventitia from Non-Aneurysmal and Aneurysmal Human Aorta
Source: PLoS One. 2014 Feb 27;9(2):e89983. doi: 10.1371/journal.pone.0089983 (PMC3937418; doi:10.1371/journal.pone.0089983)
Supplement: Figure S1 — Density of leukocyte and stromal cell subsets in the adventitia of aneurysmal and non-aneurysmal aortas. Graphical representation of the density (cells/g tissue) of (A) PMN, lymphocyte, DC, (B) macrophage, and (C) stromal cell subsets in the adventitia from non-aneurysmal (white filled symbols, n = 5) and aneurysmal (grey filled symbols, n = 5) aortas. Circles correspond to abdominal aortas, and diamond to a thoracic aorta. p values were calculated with the Mann-Whitney nonparametric test. (PDF) [file pone.0089983.s001.pdf]

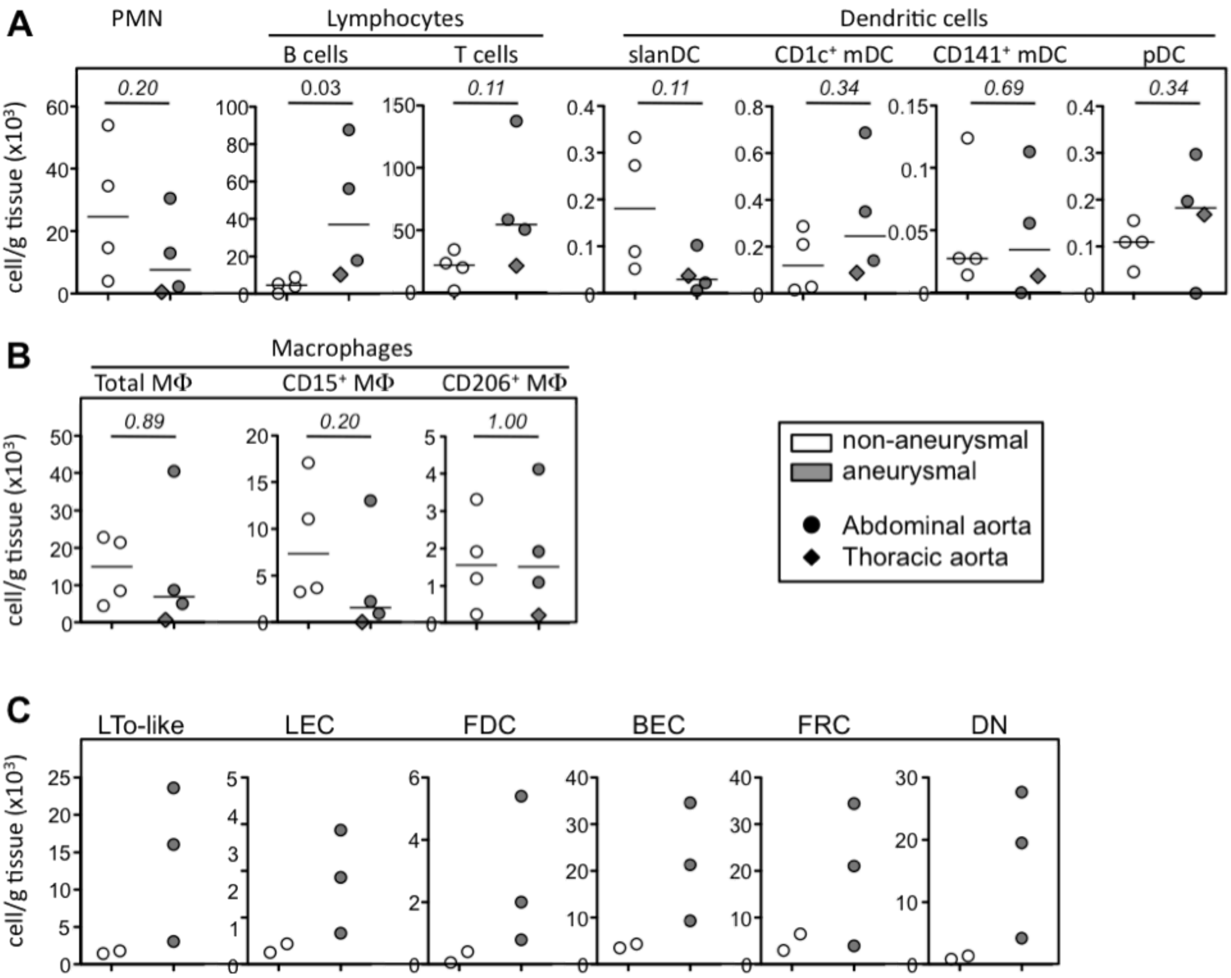

Figure S1. Density of leukocyte and stromal cell subsets in the adventitia of aneurysmal and non-aneurysmal aortas

Graphical representation of the density (cells / g tissue) of (**A**) PMN, lymphocyte, DC, (**B**) macrophage, and (**C**) stromal cell subsets in the adventitia from non-aneurysmal (white filled symbols, n=5) and aneurysmal (grey filled symbols, n=5) aortas. Circles correspond to abdominal aortas, and diamond to a thoracic aorta. p values were calculated with the Mann-Whitney nonparametric test.
