# Supplementary material for: Deciphering the Stromal and Hematopoietic Cell Network of the Adventitia from Non-Aneurysmal and Aneurysmal Human Aorta
Source: PLoS One. 2014 Feb 27;9(2):e89983. doi: 10.1371/journal.pone.0089983 (PMC3937418; doi:10.1371/journal.pone.0089983)
Supplement: Figure S2 — Comparison of the phenotype and the proliferation of all cell subsets in the adventitia from non-aneurysmal and aneurysmal aortas. (A) The gating strategy to analyze all nucleated cells (Vybrant+) and particularly stromal cell subsets from a non-aneurysmal aorta by 10-color flow cytometry is shown. (B) Comparison of the percentage among the parent population of proliferating Vybranthi cells for cell subsets defined in (Fig. 5A) in the adventitia from two non-aneurysmal (white filled symbols) and three aneurysmal (grey filled symbols) aortas. (DOC) [file pone.0089983.s002.doc]

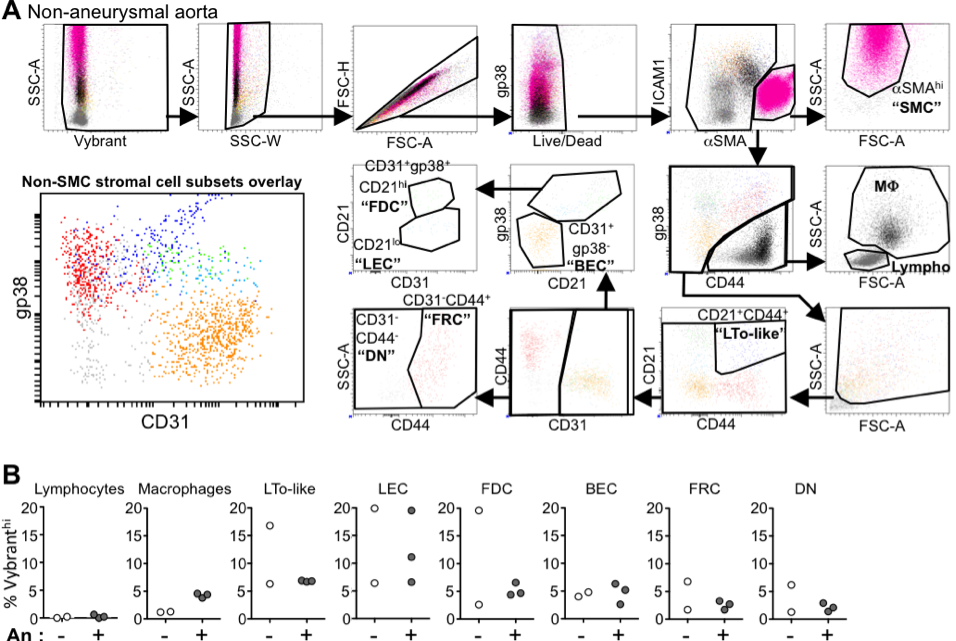


## Figure S2. Comparison of the phenotype and the proliferation of all cell subsets in the adventitia from non-aneurysmal and aneurysmal aortas

(**A**) The gating strategy to analyze all nucleated cells (Vybrant^+^) and particularly stromal cell subsets from a non-aneurysmal aorta by 10-color flow cytometry is shown. (**B**) Comparison of the percentage among the parent population of proliferating Vybrant^hi^ cells for cell subsets defined in (**Fig. 5A**) in the adventitia from two non-aneurysmal (white filled symbols) and three aneurysmal (grey filled symbols) aortas.
