# Supplementary material for: Deciphering the Stromal and Hematopoietic Cell Network of the Adventitia from Non-Aneurysmal and Aneurysmal Human Aorta
Source: PLoS One. 2014 Feb 27;9(2):e89983. doi: 10.1371/journal.pone.0089983 (PMC3937418; doi:10.1371/journal.pone.0089983)
Supplement: Table S1 — Configuration of the BD FACS LSRII used in the present study. (PDF) [file pone.0089983.s003.pdf]

Table S1. Configuration of the BD FACS LSRII used in the present study.

			Leukocyte panel		Stromal cell panel	
Laser	Band pass	Long pass	Target	Fluorochrome	Target	Flourochrome
Violet	790/20	770LP	CD19	Brilliant Violet 785	CD44	Brilliant Violet 785
(405nm)	695/40	685LP	CD3	Brilliant Violet 711		
	655/8	630LP	CD123	Brilliant Violet 650		
	605/12	595LP	CD14	Brilliant Violet 605		
	550/40	530LP	Dead cells	Live/Dead Yellow	Dead cells	Live/Dead Yellow
	500/30	495LP	CD45	V500		
	450/50		CD163	Vioblue	Proliferating cells	Vybrant Violet
Blue	780/60	755LP	CD1c	PE-Cy7	CD21	PE-Cy7
(488nm)	720/40	700LP	CD206	PercP-eFluor710	gp38	PercP-eFluor710
	670/30	655LP				
	610/20	600LP	CD15	PE-CF594		
	575/26	550LP	MerTK	PE	ICAM1	PE
	530/30	505LP	M-DC8	FITC	VCAM1	FITC
	488/10		SSC			
Red	780/60	755LP	HLA-DR	APC-H7	CD31	APC-eFluor780
(633nm)	720/40	700LP	CD16	Alexa700	αSMA	Alexa700
	660/20		CD141	APC	MadCAM1	APC
